# A Review of Evidence that Equine Influenza Viruses Are Zoonotic

**DOI:** 10.3390/pathogens5030050

**Published:** 2016-07-12

**Authors:** Tai Xie, Benjamin D. Anderson, Ulziimaa Daramragchaa, Maitsetset Chuluunbaatar, Gregory C. Gray

**Affiliations:** 1 Division of Infectious Diseases and Duke Global Health Institute, Duke University, Durham, NC 27710, USA; Tai.xie@duke.edu (T.X.); Benjamin.anderson2@duke.edu (B.D.A.); 2 Faculty of Health Service, Second Military Medical University, Shanghai 200433, China; 3 National Center for Zoonotic Diseases, Ulaanbaatar, Mongolia; ulzima_d@yahoo.com (U.D.); ch.maitsetseg@gmail.com (M.C.)

**Keywords:** equine influenza, influenza A, epidemiology, zoonotic diseases, H3N8

## Abstract

Among scientists, there exist mixed opinions whether equine influenza viruses infect man. In this report, we summarize a 2016 systematic and comprehensive review of the English, Chinese, and Mongolian scientific literature regarding evidence for equine influenza virus infections in man. Searches of PubMed, Web of Knowledge, ProQuest, CNKI, Chongqing VIP Database, Wanfang Data and MongolMed yielded 2831 articles, of which 16 met the inclusion criteria for this review. Considering these 16 publications, there was considerable experimental and observational evidence that at least H3N8 equine influenza viruses have occasionally infected man. In this review we summarize the most salient scientific reports.

## 1. Introduction

Influenza A virus-like-illnesses have been recognized in horses since at least 1299, with speculation that earlier outbreaks of equid diseases could have also been due to influenza A viruses [1,2]. A particularly well-documented influenza-like epizootic occurred among US horses during 1872, causing widespread damage to transportation and commerce [3]. Morens and Taubenberger [3] have observed that this 1872 outbreak could have been evidence of avian influenza virus cross-species infections but as the first influenza A viruses were not discovered until the 1930s, the etiology of the 1872 epizootic is unknown. Since the 1930s, only two major subtypes of equine influenza viruses (EIV) have been detected in ill horses: H7N7 (first called A/equi-1) [4] and H3N8 (first called A/equi-2) [5]. The last H7N7 viruses were isolated in the late 1970s [6,7], with only variants of H3N8 viruses causing sporadic outbreaks since then. With the exception of recent H3N8 EIV variants which have caused outbreaks in dogs [8,9], and possibly our case report of an H3N8 infection in a camel [10], recent EIV epizootics have not been associated with spill-over to non-equid species. In this report, we sought to review the English, Chinese, and Mongolian scientific literature for evidence that EIV infections have occurred in man. The Chinese and Mongolian literature were thought to be very relevant because in China’s autonomous region of Inner Mongolia and in the country of Mongolia, large numbers of horses have close contact with man.

## 2. Results

### 2.1. Search Results

**Figure 1:**
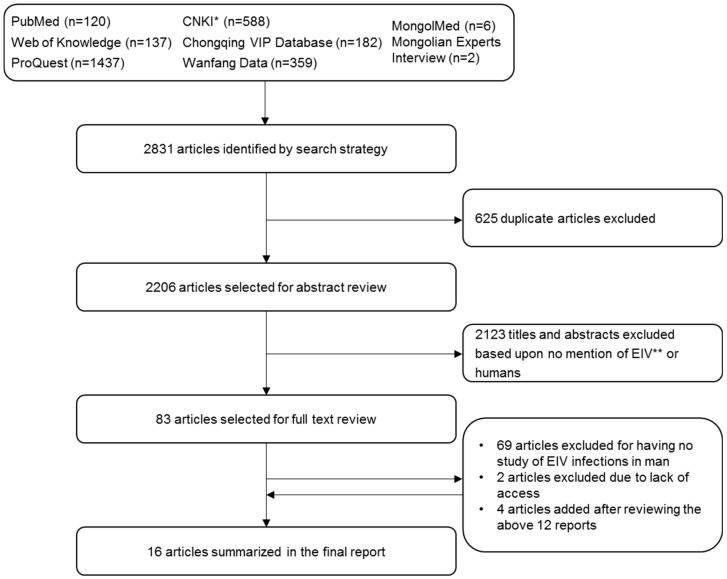
Flow diagram of the literature search process. By the search strategy, a total of 2831 articles were identified which was comprised of 1694 English language articles, 1129 Chinese, 7 Mongolian, and 1 Russian report. Duplicate articles were removed. See text for more details. * CNKI = Chinese National Knowledge Infrastructure; ** EIV = Equine Influenza Virus.

We identified 2831 articles using multiple search techniques (Figure 1). Six hundred and twenty-five duplicates were then removed. The abstracts of the resultant 2206 articles (1405 English language articles, 793 Chinese, 7 Mongolian, 1 Russian) were reviewed (TX and BA) and after careful consideration were reduced to 83 unique reports. These full articles were next retrieved and carefully read (TX). Examination of the references cited by the 83 reports yielded 4 more articles. After full text review, 71 of the articles were excluded because there was no mention of human infection. The resultant 16 publications (all in English) were included in this review (Table 1).

### 2.2. Historial Evidence of EIV Infections in Man

In this review we found considerable historical evidence of EIV infections in man. Although they are careful to explain confounders, Morens and Taubenberger [2] document numerous such historical observations. They found that from 1658 to the early 20th century, EIV outbreaks in horses often occurred 3 weeks or so before human influenza-like-illnesses (ILIs). In particular, scholars have implicated the 1889 human pandemic as likely caused by a H3N8 EIV [25,26,27]. Serological studies, published in the 1960s, of people who lived during that 1892 era, are most compelling in documenting elevated antibodies against H3N8 EIV [14,17].

### 2.3. Human Volunteer H3N8 EIV Experimental Infection

Several healthy human EIV challenge experiments were conducted in the 1960s. In 1965, hospitalized volunteers received challenges with live equine H3N8 virus (A/Equi-2/Miami/63) and were carefully monitored for evidence of infection. Five healthy adult volunteers each received 2.5 mL of undiluted equine H3N8 inoculum (the A/Equi-2/Miami/63 virus strain was serially passaged 5 times in embryonated hens’ eggs and twice in primary hamster kidney culture), among which 1 mL was administered by pipette directly into the nasal cavities and 1.5 mL nebulized into the nasal cavities and oropharynx [13]. Each subject had viable virus isolated 3 days after challenge, but only one subject developed clinical signs (rectal temperature of 39 °C, mild sore throat, nasal congestion and obstruction, and leukocytosis of 9500). In 1966, Alford et al. conducted another human volunteer challenge study with H3N8 EIV [18] using the same methods as Kasel et al. Four (12.1%) of 33 volunteers developed clinical signs with one developing an oral temperature of 39.4 °C, 21 (63.6%) volunteers had virus isolated 3 days after inoculation, and 20 (60.6%) developed 4-fold or greater increases in antibody (as measured by hemagglutination-inhibition and hemadsorption-inhibition neutralization assays) 28 days post-inoculation. Kasel et al. performed another human challenge experiment in 1969 [19,28]. Fifteen adult male volunteers (had low or no detectable titers to human H3N2 or A2/Hong Kong/68 viruses) received nasopharyngeal nebulization challenges with 10^6.75^ TCID_50_ equine H3N8 virus. Thirteen (86.7%) of 15 volunteers developed signs of illness, and virus was cultured from samples obtained from all volunteers at day 4 post-inoculation. Systemic illness and febrile upper respiratory illness were the most common clinical signs. The experimental data suggested that the H3N8 virus was not attenuated following passage in humans, as it was still capable of infecting and causing illness in horses.

### 2.4. Evidence of Human EIV Infection after Infected Equid Exposure

While there are scattered reports in the news media and historical science writings that horses, dogs, humans, and even cats concomitantly developed ILIs [1,2,29], modern documentation of human EIV infection in observational reports is sparse.

The country of Mongolia has the largest horse-to-man population ratio in the world and has often suffered large epizootics of EIV [30]. For instance, a 1983-4 H3N8 epizootic affected an estimated 891,000 horses causing 176,000 deaths [30]. After anecdotal reports suggested children in Mongolia developed respiratory illnesses after exposure to horses with EIV infection, we conducted a prospective cohort study to investigate human infections with EIV. From 2009 to 2011 we enrolled and followed 439 Mongolian adults [20,21], many with occupational exposure to horses, for evidence of EIV infection. One hundred subjects developed an ILI and were investigated with molecular and viral culture studies for influenza. Thirty-six ILI cases (36%) were identified as influenza A infections by qRT-PCR but none had evidence of EIV. Examination of sera upon enrollment, and at 12 and 24 months later, revealed that approximately 40 participants had detectable microneutralization antibody titers (>1:10) against A/Equine/Mongolia/01/2008(H3N8). However, all such titers were <1:80 (accepted benchmark for acute infection) and evidence was not compelling for recent EIV infection.

Soon after, at least 1400 horses were infected with H3N8 EIV during a 2007 epizootic in New South Wales and Queensland, Australia. We conducted a cross-sectional study for EIV infections among 89 people exposed to those sick horses (and 11 controls) [22]. Serum samples were tested for the presence of antibodies against H3N8 EIV using hemagglutination, microneutralization, and enzyme-linked lectin assays. Again, evidence of acute EIV infection was sparse, with only 9 study participants having low titers of antibodies which might easily have been associated with cross-reacting antibody from human influenza virus or vaccines [24].

More recently, we reported results from our 2005 cross-sectional study of 94 horse-exposed adults and 34 non-exposed controls from three sites in the US state of Iowa. Employing three different types of assays against two strains of H3N8 and one strain of H7N7 EIV, data suggested that at least a portion of horse-exposed adults had been previously infected with an A/equine/Ohio/2003(H3N8)-like strain. Eleven (11.7%, maximum titer 1:320) horse-exposed adults and two (5.9%, maximum titer 1:160) control subjects had microneutralization titers ≥1:80. Among the horse-exposed adults, 18 (19.1%) were positive by a novel neuraminidase inhibition assay and eight (8.5%) had elevated enzyme-linked-lectin assay titers ≥1:10. In the biostatistical analyses, work as an equine veterinarian was associated with increased seroreactivity.

## 3. Discussion

At least three H3N8 EIV human challenge studies [13,18,19] clearly document that human infection is possible with H3N8 EIV and that subjects may not manifest signs of illness. Also important were the 1960s observations (before molecular viral characterization was available) that human-passaged viruses demonstrated no evidence of attenuation.

While the observational studies of human EIV infection in Mongolia [20,21] and Australia [22] were essentially negative, the 2005 cross-sectional study of horse-exposed adults in Iowa [24] was more compelling as observational evidence for human infection with EIV. In this later work, elevated microneutratlization (MN) titers (≥1:80) among the horse-exposed were clearly associated with having a positive novel neuraminidase inhibition (NI) assay (OR = 4.9; 95% CI = 1.3–18.7). There was also a significant association between elevated enzyme-linked lectin (ELLA) titers (≥1:10) and a positive NI assay (OR = 53.2; 95% CI = 5.9–478.5). The ELLA assay is gaining popularity, and may be more specific and easier to perform than the MN assays. The N8 antigen used in the ELLA assay was obtained from BEI resources [31].

During our review we became aware that there have been a number of non-equine influenza A viral segments identified in horses. In searching the Influenza Research Database (IRD), we found that genome segments related to influenza A H1N8, H5N1, H7N1 and H9N2 strains have all been detected among samples collected from horses. Although these identifications seem to be rare, accounting for less than 0.5% of all EIV detections, it may indicate that horses are at least susceptible to more diverse strains of influenza A virus than previously thought. For example, in 2011 a novel H9N2 influenza A virus was detected, isolated, and fully sequenced in a study of horses in Guangxi Province, China [32]. 

Additionally interesting findings are that equids are not always H3N8 EIV “dead-end” hosts. H3N8 EIVs have been detected in dogs in USA, UK and Australia [5,9,33,34,35], in pigs and cats in China [29,36], and in a camel in Mongolia [10]. These data support the need for continued surveillance among equids for novel influenza A viruses.

Even with the experimental and observational data supporting evidence that H3N8 EIVs may infect man, we must be careful to temper these findings with the knowledge that serological assays against zoonotic influenza, such as equine H3N8, may be confounded by non-specific assay inhibitors and pre-existing antibodies against other influenza strains such as those caused by other human H3 viruses or vaccine.

## 4. Materials and Methods

In February and March 2016, we searched English, Chinese and Mongolian literature databases, as well as queried senior scientists in Mongolia for relevant literature. English-based databases included: PubMed, Web of Knowledge and ProQuest. Chinese-based databases included: Chinese National Knowledge Infrastructure (CNKI), Chongqing VIP Database and Wanfang Data. Similarly, MongolMed was queried for related Mongolian citations. The following search query was used for PubMed, Web of Knowledge, and ProQuest databases: (“equine influenza” OR “horse influenza” OR “H3N8” OR “H7N7”) AND (“human infection” OR zoono*). The following search query was used for CNKI, Chongqing VIP Database, and Wanfang Data: SU = ‘Maliugan’ OR SU = ‘Maliuxingxingganmao’ OR SU = ‘equine influenza’ OR SU = ‘horse influenza’ OR SU = ‘H3N8’ OR SU = ‘H7N7’. The search terms “human infection” OR zoono* were not included when searching the Chinese literature databases, as the number of articles identified when using these terms was very low. Chinese papers identified with equine influenza search terms were instead manually reviewed to select papers that also included “human infection” or zoono*. We searched MongolMed for citations using “equine influenza” OR “horse influenza” OR “адууны томуу”. Mongolian influenza experts were also interviewed for their knowledge of related reports.

## 5. Conclusions

The historical, observational, and experimental data are compelling in supporting the premise that EIV infections occasionally occur in man. While in recent years, human infections with EIVs have not often been associated with signs of infection, the propensity for influenza A viruses to change makes these viruses worthy of our attention. In particular, should H7N7 EIV strains again emerge, most humans would have little cross-reacting antibody, and the threat to humans might be quite different than that commonly seen today for H3N8. We believe these observations support close surveillance for novel influenza virus emergence among equids.

## Figures and Tables

**Table 1:** Publications found to be important in considering equine influenza virus infections in man.

Publications	Country and Year	Main Summary
Gaidamaka et al. [11]	Ukraine 1959	The authors present horse serological data supporting a position that an EIV epizootic among race horses may have been associated with a temporally-related recent human influenza epidemic.
Marois et al. [12]	Canada 1963	The authors report an EIV epizootic among 800 horses with an 86% attack rate. The authors report viral culture and serological evidence that virus came from humans and speculate such EIV outbreaks in horses may put humans at risk.
Kasel et al. [13]	USA 1965	First NIH experimental infection study of five human volunteers with H3N8 equine influenza virus. One (20%) developed signs and symptoms.
Minuse et al. [14]	USA 1965	Cross-sectional seroepidemiological study of 300 humans for antibodies against EIV H3N8 equine strains suggesting different exposure by age group during 1870 to 1900.
Schild et al. [15]	UK 1965	The authors summarize a series of serological studies of people of various ages and illness states. Persons >65yrs had neutralizing antibodies against A/Equine/Miami/63 (later determined to be H3N8 or Equi-2)
Voth et al. [16]	USA 1966	Serological study of 119 study subjects found 34% with slightly elevated antibody against a 1963 H3N8 equine virus.
Masurel et al. [17]	Netherlands 1966	Cross-sectional seroepidemiological study of 2750 humans suggested many had exposure to a H3N8 EIV during the period 1896–1900.
Alford et al. [18]	USA 1967	Second NIH experimental infection study of 33 human volunteers with H3N8 equine influenza virus: 63% had viable virus after 3 days, 60% seroconverted, and 12% had clinical signs or symptoms.
Kasel et al. [19]	USA 1969	A third NIH report of experimental infection with H3N8 EIV in 15 human volunteers: 100% had viable virus after 4 days, 93% developed a 4-fold rise in antibody, and 87% developed signs of illness.
Morens et al. [2]	USA 2010	Historical review of evidence that EIV may have infected man.
Morens et al. [3]	USA 2010	Suggestion that the 1872 equine influenza epizootic may have been associated with a temporally-related human influenza epidemic.
Khurelbaatar et al. [20]	Mongolia 2013	A cross-sectional study of 439 adult Mongolians revealed some elevated antibodies against H3N8 EIV, but it was not clearly related to horse exposure.
Khurelbaatar et al. [21]	Mongolia 2014	Prospective study of 439 Mongolians for evidence of EIV infections. Sparse serological evidence of elevated titers against H3N8 EIV.
Fiona et al. [22]	Australia 2014	Eighty-nine humans exposed to a 2007 Australian H3N8 EIV horse epizootic had little evidence of EIV infection.
Parrish et al. [23]	USA 2015	Recent review of equine and canine influenza literature. Possible threat to humans discussed.
Larson et al. [24]	USA 2015	Serological evidence supported the premise that occupational exposure to horses may lead to human infection with H3N8 EIV.

## Abbreviations

The following abbreviations are used in this manuscript:

CNKI	Chinese National Knowledge Infrastructure
EIV	Equine Influenza Virus
ILI	Influenza-like-illness
IRD	Influenza Research Database
TCID_50_	50% tissue culture infective doses
UK	United Kingdom
USA	United States of America
